# Automated detection of gallbladder stones using a deep learning algorithm on computed tomography scans

**DOI:** 10.1007/s00261-025-05352-6

**Published:** 2026-01-12

**Authors:** Manfred T. Meyer, Jakob Wasserthal, Joshy Cyriac, Shan Yang, Elmar M. Merkle, Tobias Heye

**Affiliations:** Department of Radiology and Nuclear Medicine, University Hospital of Basel, Basel, Switzerland

**Keywords:** Gallbladder, Cholecystolithiasis, Deep learning, Sensitivity and specificity, CT

## Abstract

**Purpose:**

To develop and evaluate the diagnostic accuracy of a deep learning algorithm for automated gallstone detection on CT.

**Methods:**

This retrospective single-center study included randomly selected CT scans from January 2018 to June 2019: 493 gallstone-positive, 470 gallstone-negative, and 180 post-cholecystectomy scans according to the original report (reference standard). Deep learning models were trained on segmented gallbladders (452 scans) and gallstone bounding-box labels (400 scans), randomly selected and manually labeled. All models used 5-fold cross-validation. Gallstone characteristics were collected (e.g., quantity, size, conspicuity). Where available, ultrasound and MRI reports within six months of CT were reviewed for cholecystolithiasis. Diagnostic performance was evaluated on a test set of 90 scans (30 per category). Statistical analyses included Dice coefficient, sensitivity, specificity, and ROC analysis.

**Results:**

Ninety patients (mean age 64.9 ± 17.9 years; 46 women) were evaluated. The segmentation algorithm achieved a median Dice score of 94.4% (IQR 4.5%). The detection pipeline identified gallstone-positive scans with a sensitivity of 96.7% (95% CI: 90.2–100.0) and specificity of 83.3% (95% CI: 70.0–96.7). Sensitivity was highest for hyperdense (97.3%) and large stones (>10 mm, 97.1%) but lower for gaseous (71.4%), isodense (78.6%), and small stones (<5 mm, 88.9%). Cholecystectomy was correctly identified in 76.7% of cases. Gallstone presence correlated with larger gallbladder volumes (mean 42.8 mL ± 38.3 vs. 24.0 mL ± 20.7, p < 0.001). Ultrasound/MRI comparisons (available for 178 [18.5%] of CTs with a gallbladder) showed low false-positive (0.6%) and false-negative (4.5%) rates for CT.

**Conclusion:**

The algorithm detects gallstones in CT scans with high sensitivity, particularly for large, conspicuous stones.

## Introduction

Gallstone disease is very common in the Western world with an estimated prevalence of 10–20% in European and American adults [1]. Approximately one in five gallstone carriers experiences gallstone-related events over a 20 year period [2]. A 2014 study identified cholelithiasis as one of the two most common principal diagnoses involving the gastrointestinal tract in US hospitals, leading to significant socioeconomic costs [3]. Gallstone disease is associated with an elevated risk for gastrointestinal cancer [4] and the presence of multiple and small stones increases the risk for pancreatitis, which carries a significant mortality rate [5]. Therefore, early and reliable detection of gallstones is of clinical importance.

Ultrasonography is the method of choice for the diagnosis of cholecystolithiasis with a reported sensitivity of 84–85% and specificity of 99–100% [6, 7]. Comparatively, computed tomography (CT) has a lower sensitivity (79%) but similar specificity (99%) [6]. The lower sensitivity of CT is mostly due to similar density values of gallstones with a high cholesterol content compared to the surrounding bile. Conversely, higher calcium content of a gallstone is correlated with higher conspicuity [8, 9]. In a German study, over 90% of gallstones were primarily composed of cholesterol, which is thought to be linked to the Western diet and the high prevalence of metabolic syndrome [10].

Several large studies on incidental findings in CT scans of mostly trauma patients in the US and Europe report gallstones in 0.4–4.2% of all patients [11–17]. This appears low given the 10–20% prevalence of gallstones [1] and a CT sensitivity of approximately 79% [6]. We therefore hypothesize that the presence of gallstones on CT is generally underreported. An assistant in the form of a clinical decision support system (CDSS), which alerts radiologists to positive algorithmic gallstone detections, may help reduce underreporting and false negatives.

The aim of this study is to develop and evaluate the diagnostic accuracy of a fully automated and autonomous deep learning algorithm for detecting gallstones using standard deep learning technology. The output should be summarized in a report designed to be helpful to both radiologists and clinicians.

## Materials and methods

Data were retrospectively collected from our single-center hospital databases. The study was approved by the local ethics committee (EKNZ, project 2019-01989), with informed consent waived.

### Case selection

CT scans from January 2018 to June 2019 were searched using “PACS/RIS Crawler” (version 1.3.0) [18]. At our institution, all standard abdominal CT scans are reported using structured templates in which the presence or absence of gallstones is explicitly documented. All examinations were read independently by at least two physicians. A board-certified, fellowship-trained abdominal imaging specialist approved all reports. CT scans with reports indicating the presence (500 scans) or absence (500 scans) of cholecystolithiasis were identified. An additional 200 scans from post-cholecystectomy patients were collected. These target numbers were chosen based on experience from previous, similar projects. The ground truth label for each scan was derived solely from the original radiology report (reference standard) and no additional image review was performed. Selection of suitable cases was conducted between November 2019 and March 2020. Examinations were randomly included until the target count for each subgroup was met. The following inclusion criteria had to apply: CT scan coverage of the complete biliary system, intravenous administration of a radiocontrast agent, imaging during the portal venous phase, and availability of a series with 5 mm slice thickness. Exclusion criteria were defined as: mention of cholecystitis or malignant gallbladder pathologies in the report, or strong artifacts impairing gallbladder interpretability. During case selection, only the final report was visible, and no images were displayed. Beyond age and sex, no additional patient characteristics were collected.

All examinations were performed on one of the following CT scanners by Siemens Healthineers (Forchheim, Germany): Somatom Definition Edge, Somatom Definition Flash, Somatom Definition AS+.

### Data labelling

The 1200 selected CT examinations were transferred to “Nora”, a web-based framework for medical image analysis [19]. Some scans were excluded from further analysis, primarily due to transmission or processing errors (e.g. missing slices), incomplete depiction of the gallbladder, or strong image artifacts reducing gallbladder interpretability. For further analysis, 493 gallstone-positive scans, 470 gallstone-negative scans, and 180 scans from post-cholecystectomy patients were included.

For 963 CT volumes with a gallbladder, a short single-choice questionnaire was completed by M.T.M. (resident).

452 CT examinations featuring a gallbladder were randomly selected and the gallbladder was manually segmented by M.T.M. In instances of uncertainty, a flag was set, and the case was reviewed by T.H. (a board-certified and fellowship-trained abdominal imaging specialist). Based on prior experience with comparable annotation efforts, this sample size was considered sufficient for segmentation model training, with diminishing returns expected from additional cases.

Using these manual gallbladder segmentations, axial slices from a random subset of 400 CT scans showing any part of the gallbladder were extracted using an abdominal window (W 350; L 50). On these grayscale images, both the gallbladder and any gallstones were labelled with rectangular bounding boxes. Clearly separable gallstones received separate bounding boxes, while poorly separable clusters of gallstones were grouped together in a single bounding box.

### Datasets

**Fig. 1:**
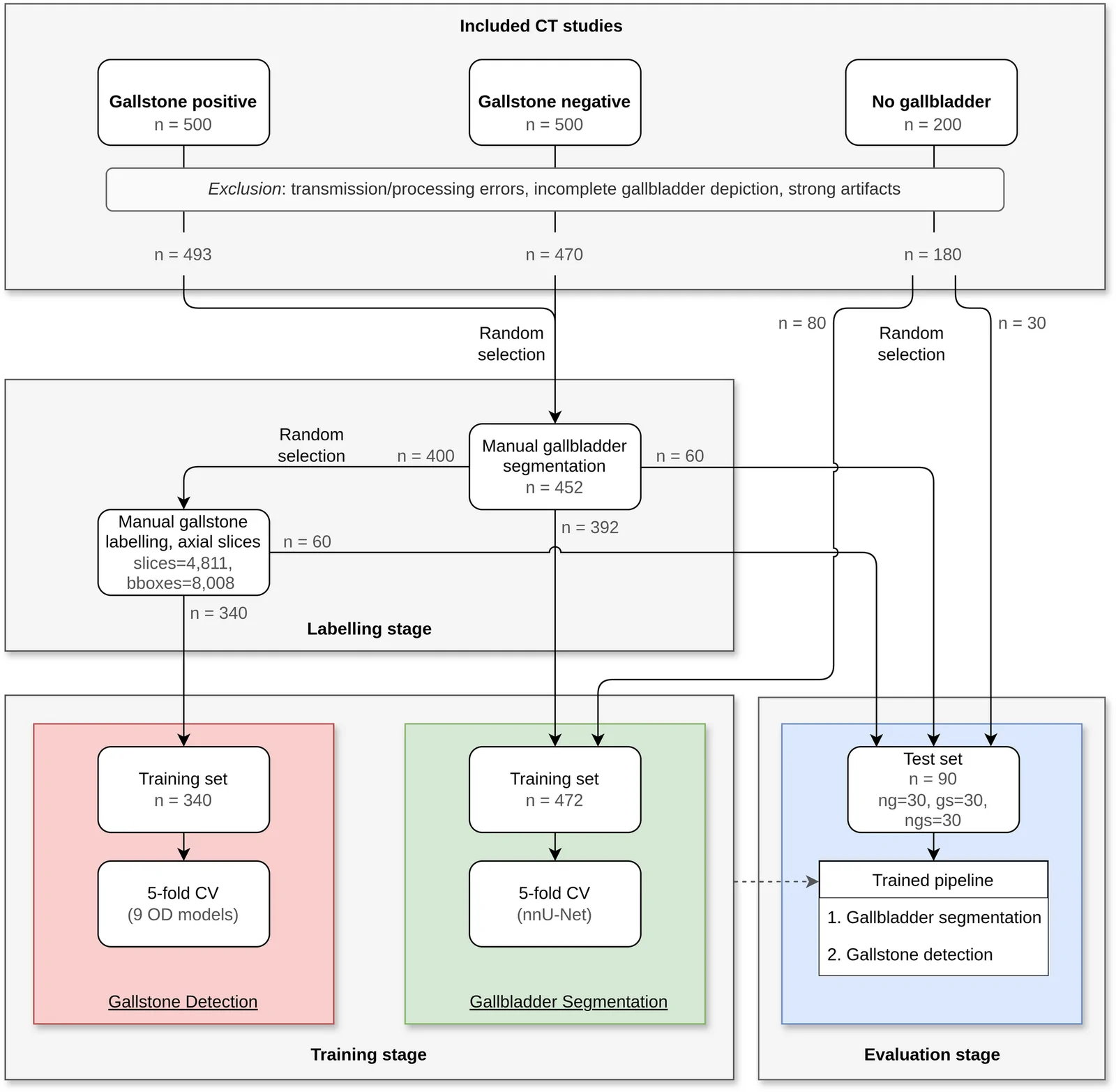
Flow of CT scans from selection to training and evaluation datasets. Segmentation and detection algorithms were trained separately with partially overlapping training datasets. The end-to-end pipeline was then evaluated on a separate test set. n number of CT scans, CV cross-validation, OD object detection, bboxes bounding boxes, ng no gallbladder, gs gallstones, ngs no gallstones

All models were trained using fivefold cross-validation. Cross-validation splits were grouped by patient ID to prevent images of the same patient (or study) from appearing in both training, validation or test data. The final end-to-end pipeline was evaluated on a separate test set with 30 scans from each of the following three categories: gallstones, no gallstones, no gallbladder. Figure 1 illustrates the data flow, including the manual labelling and segmentation processes as well as the training and evaluation stages of the deep learning models.

### Gallbladder segmentation algorithm

Algorithmic gallbladder segmentation was performed using a two-step process. First, the entire CT image was cropped to a region around the liver using an nnU-Net-based segmentation model (TotalSegmentator) [20]. Second, these liver crops, along with manual gallbladder segmentations were used to train another nnU-Net model specifically for gallbladder segmentation [21].

### Gallstone detection algorithm

Several deep learning models were trained on the extracted axial images with bounding-box labels to detect the gallbladder and gallstones. These object detection models were based on either RetinaNet with a ResNet101 backbone [22–24], or EfficientDet [25, 26] with ResNet50 [24] or EfficientNet D4 [27] backbones. All models were initially pretrained on diverse real-world images, enhancing both accuracy and training convergence [28, 29].

The models processed input images along with their ground truth labels using one of three data loaders. The first loaded a single slice as an RGB grayscale image (with identical color channels). The second loader stacked three consecutive axial slices into the three RGB channels to provide spatial context (termed “multi-slice images”). The third loader randomly pasted a gallstone from another slice (from any patient in the training data) into the gallbladder area of the current image, applying extensive image augmentation to the pasted stones. Additionally, random image augmentations, including adjustments to brightness, contrast, translation, scaling, and rotation, were applied to all training images to enhance model robustness.

A total of nine object detection models (3 backbones x 3 data loaders) were fine-tuned using image inputs generated by the different data loaders, training over 24 epochs using a learning rate of 0.001 and batch size of 4 on a single graphics processing unit (GPU). The exponential moving average (EMA) model achieving the highest validation score was selected for each training fold.

### Deep learning inference

**Fig. 2:**
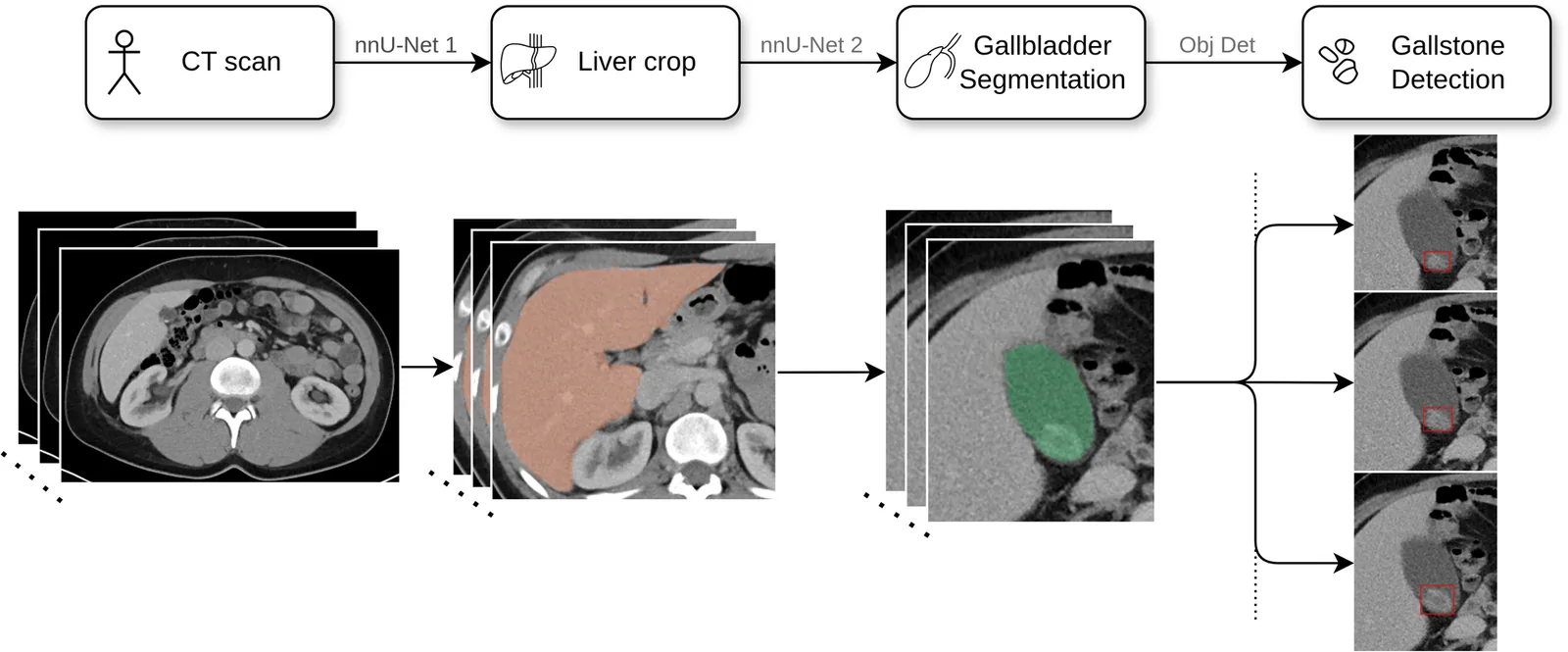
Inference pipeline with its stages: (1) liver segmentation on the input CT scan using an nnU-Net, (2) cropping to the liver region to decrease the search space, (3) segmentation of the gallbladder in the liver crop using a second nnU-Net, (4) detection of gallstones using an ensemble of several object detection (Obj Det) deep learning models on the axial slices

For inference, the following pipeline was applied (illustrated in Fig. 2): First, the entire CT scan was cropped to the liver region. Second, this crop was processed by the gallbladder segmentation algorithm. Third, the detection algorithms were applied to all axial slices showing the gallbladder. Fourth, the gallstone bounding box predictions from the nine object detection models were ensembled using the Weighted Boxes Fusion technique [30]. Fifth, gallstone bounding boxes were discarded if they overlapped with the gallbladder segmentation from the second step by less than 30% of their area or if their confidence value was below a determined cutoff value. Sixth, a scan was deemed gallstone positive if any bounding boxes remained post-processing.

### Correlation with US and MRI studies

For all selected cases with a gallbladder, hospital imaging databases were searched for ultrasound (US) and magnetic resonance imaging (MRI) examinations performed within six months of the index study. Both US and MRI have higher diagnostic accuracy than CT for detecting gallstones. Therefore, if the report from either a US or MRI examination conflicted with the CT report, the diagnosis from the former took precedence. In such cases, the CT was marked as a false positive or false negative, respectively.

### Evaluation

Segmentation performance was evaluated using the Sørensen–Dice coefficient, calculated as Dice $=\frac{2\left|\mathrm{S}\cap\mathrm{G}\right|}{\left|\mathrm{S}\right|+\left|\mathrm{G}\right|}$, where *S* is the segmentation result, *G* is the ground truth, and ∣⋅∣ denotes cardinality.

Data distribution normality was assessed via histograms and quantile-quantile plots. Intergroup differences were analyzed using the Mann-Whitney U test for non-normal distributions. A *p* value < 0.05 was considered statistically significant (α = 0.05).

Object detection performance was measured by the mean average precision score (mAP), as per the “Common Objects in Context” (COCO) dataset standards, using the pycocotools package [29]. The reported mAP corresponds to the AP value at an intersection over union (IoU) threshold of 0.5 [31].

Optimal cutoff values for bounding box confidence scores were determined using receiver operating characteristic (ROC) curves and Youden’s index on the validation splits. Diagnostic performance was evaluated by discarding bounding boxes with confidence scores below the cutoff.

Gallstone and cholecystectomy detection metrics were reported on a per-scan basis. Sensitivity for different gallstone subgroups were assessed on the validation splits of the training data using fivefold cross-validation after discarding gallstone bounding boxes that did not overlap with any gallbladder bounding box. Sensitivity and specificity were reported with 95% confidence intervals calculated using a normal (Gaussian) approximation to the binomial distribution, implemented with Python’s statsmodels package (version 0.14.2).

## Results

### Demographics

Limited demographic data were collected as shown in Table 1. The gallbladder segmentation algorithm was trained using 472 CT scans (48.9% women, mean age 68.0 ± 14.2 years). The gallstone detection algorithm training involved 340 CT scans (45.3% women, mean age 69.4 ± 12.9 years). The comprehensive evaluation of the end-to-end algorithm, combining segmentation and gallstone detection, included 90 CT scans (51.1% women, mean age 64.9 ± 17.9).

**Table 1:** Demographic characteristics of the training and evaluation datasets

	Gallbladder segmentation training set	Gallstones detection training set	Evaluation/test set
Total no.	472	340	90
No. women	231 (48.9%)	154 (45.3%)	46 (51.1%)
Age (y)	68.0 (± 14.2)	69.4 (± 12.9)	64.9 (± 17.9)

Data are numbers of CT scans, with percentages in parentheses. Age is reported as means ± SD

### Segmentation algorithm

On the test set of 60 CT examinations containing gallbladders, the gallbladder segmentation algorithm achieved a median Dice score of 94.4% (interquartile range [IQR], 4.5%). A notable outlier was one CT examination where the Dice score was 0, indicating no overlap with manual segmentation.

Among 30 subjects from the test set who underwent cholecystectomy, the algorithm correctly predicted a gallbladder volume of 0 mL in 15 cases (50%). The median predicted volume was 0.1 mL (IQR 1.7 mL). Using a cutoff of 2 mL, based on the 1st percentile of 392 manually segmented gallbladder volumes from the training set, the algorithm correctly classifies 23 of 30 scans (76.7%) as post-cholecystectomy. One scan from the test set with a gallbladder had a predicted gallbladder volume of 1.6 mL and was therefore erroneously classified as having no gallbladder. Overall, the cholecystectomy detection subtask reached a sensitivity of 76.7% (95% CI 61.5–91.8) and specificity of 98.3% (95% CI 95.1–100.0) on a per-scan basis when evaluated on all 90 test set scans.

### Gallbladder volumes

**Fig. 3:**
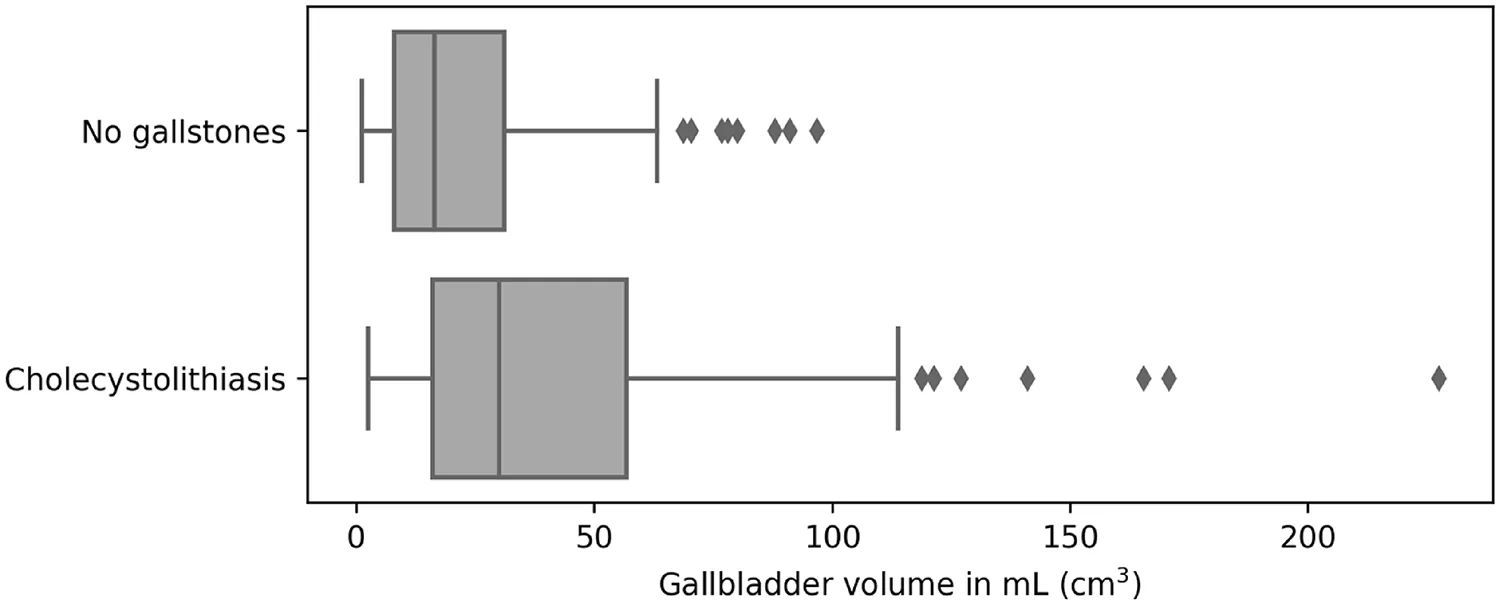
Gallbladder volumes of 452 manually segmented CT scans (254 with gallstones, 198 without gallstones). Median volume for gallstone negative cases is 17.8 mL (IQR 26.5 mL) and 30.9 mL (IQR 41.0 mL) for gallstone positive individuals. Box plot whiskers span the width of 1.5 IQR, outliers are shown as diamonds

A statistically significant difference in gallbladder volumes was observed across 452 manually segmented CT scans (*p* < 0.001). Subjects with gallstones had a mean volume of 42.8 ± 38.3 mL (254 scans), compared to 24.0 ± 20.7 mL (198 scans) in patients without gallstones (Fig. 3).

### Gallstone detection

Processing a complete CT scan, from raw input file to final report including gallbladder volume and gallstone detection results, typically takes around 30 s using a consumer-grade GPU (NVIDIA GeForce RTX 4090).

The end-to-end pipeline on the test set (Table 2) correctly identified 29 out of 30 gallstone-positive scans (sensitivity 96.7%, 95% CI 90.2–100.0) and 25 out of 30 gallstone-negative scans (specificity 83.3%, 95% CI 70.0–96.7) on a per-scan basis. The area under the receiver operating characteristic curve (AUROC) on a per-scan basis was 0.977.

**Table 2:** Diagnostic performance of the algorithm assessed on the test set on a per-scan basis

	Cases	Metric (95% CI)
Sensitivity	29/30	96.7% (90.2–100.0)
Specificity	25/30	83.3% (70.0–96.7)
Accuracy	54/60	90.0% (82.4–97.6)

The mean average precision (mAP@0.5), which is often used to evaluate object detection algorithms, was 81.7% in the validation set, calculated from the combined outputs of nine trained models without additional post-processing.

Table 3 provides an overview of the algorithm’s performance across various gallbladder and gallstone characteristics as assessed by a human. Generally, the algorithm shows high sensitivity (> 97%) with large, hyperdense and easily visible gallstones. However, sensitivity decreases for gaseous (71.4%) or isodense stones (78.6%), collapsed gallbladders (75%), and stones with low conspicuity (38.1%).

**Table 3:** Performance characteristics of the algorithm regarding different parameters as assessed by a human on 215 gallstone positive scans

		Sensitivity (%)	(95% CI)	TP	Total
Gallbladder	Collapsed	75.0	(50.5–99.5)	9	12
	Normal/distended	93.0	(89.5–96.6)	187	201
Gallstone quantity	One	89.8	(82.1–97.5)	53	59
	Few (2–5)	92.4	(86.6–98.2)	73	79
	Many (> 5)	93.5	(88.0–99.0)	72	77
Gallstone size	Small (< 5 mm)	88.9	(82.7–95.1)	88	99
	Medium (5–10 mm)	91.5	(83.5–99.5)	43	47
	Large (> 10 mm)	97.1	(93.1–100.0)	67	69
Gallstone type	Hyperdense	97.3	(94.6–99.9)	142	146
	Isodense	78.6	(66.2–91.0)	33	42
	Hypodense	88.9	(74.4–100.0)	16	18
	Very hypodense (gas)	71.4	(38.0–100.0)	5	7
Gallstone visibility	Easy	98.7	(96.9–100.0)	149	151
	Moderate	95.2	(88.8–100.0)	40	42
	Hard	38.1	(17.3–58.9)	8	21
Total	All positive scans	92.1	(88.5–95.7)	198	215

Evaluation is performed on a per-scan basis on the validation splits using fivefold cross-validation. Specificity is 92.8% (95% CI 88.3–97.3) for all

*CI* confidence interval, *TP* true positives

**Fig. 4:**
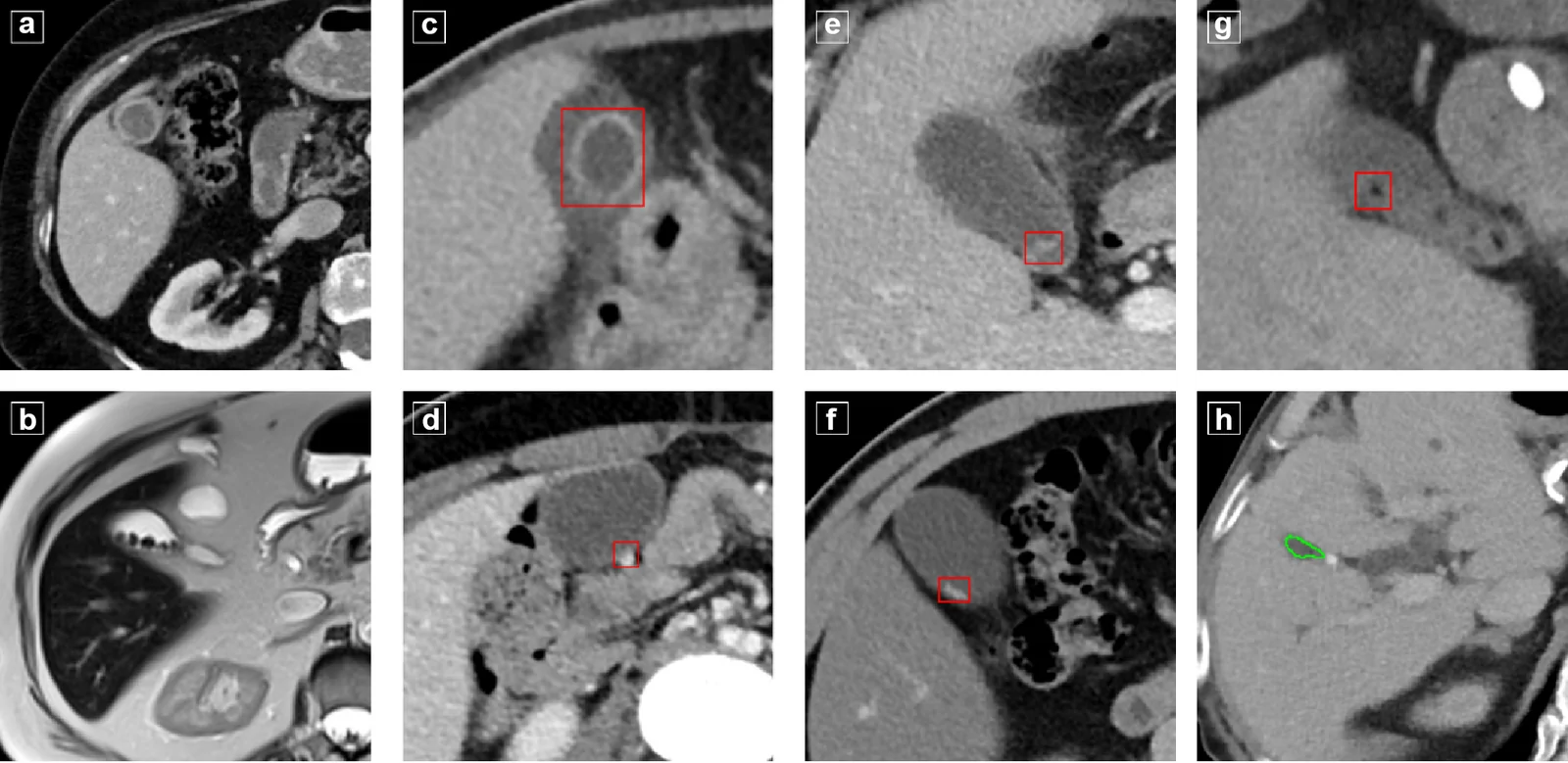
Samples from the test set using the trained end-to-end algorithm. **a** False-negative case: The gallstones in the CT were not recognized by the algorithm. **b** On a T2-weighted MR image (performed 50 days after the CT scan), the gallstones are clearly visible. **c** Gallstone negative CT study with fluid in the gallbladder fossa reactive to duodenitis. The algorithm misclassified the enhanced gallbladder wall as a gallstone. **d** False-positive detection of a blood vessel adjacent to the gallbladder wall. **e** False-positive detection of a spiral valve of Heister in the gallbladder infundibulum. **f** True-positive sample of small gallstones along the posterior wall in the gallbladder fundus. **g** Gaseous/hypodense stones are generally poorly recognized by the algorithm (only one of multiple stones detected). **h** CT of a patient who underwent cholecystectomy where the algorithm segmented a small fluid collection in the gallbladder fossa (0.7 mL). Since its volume was below the threshold of 2 mL, the scan was correctly classified as post-cholecystectomy

**Fig. 5:**
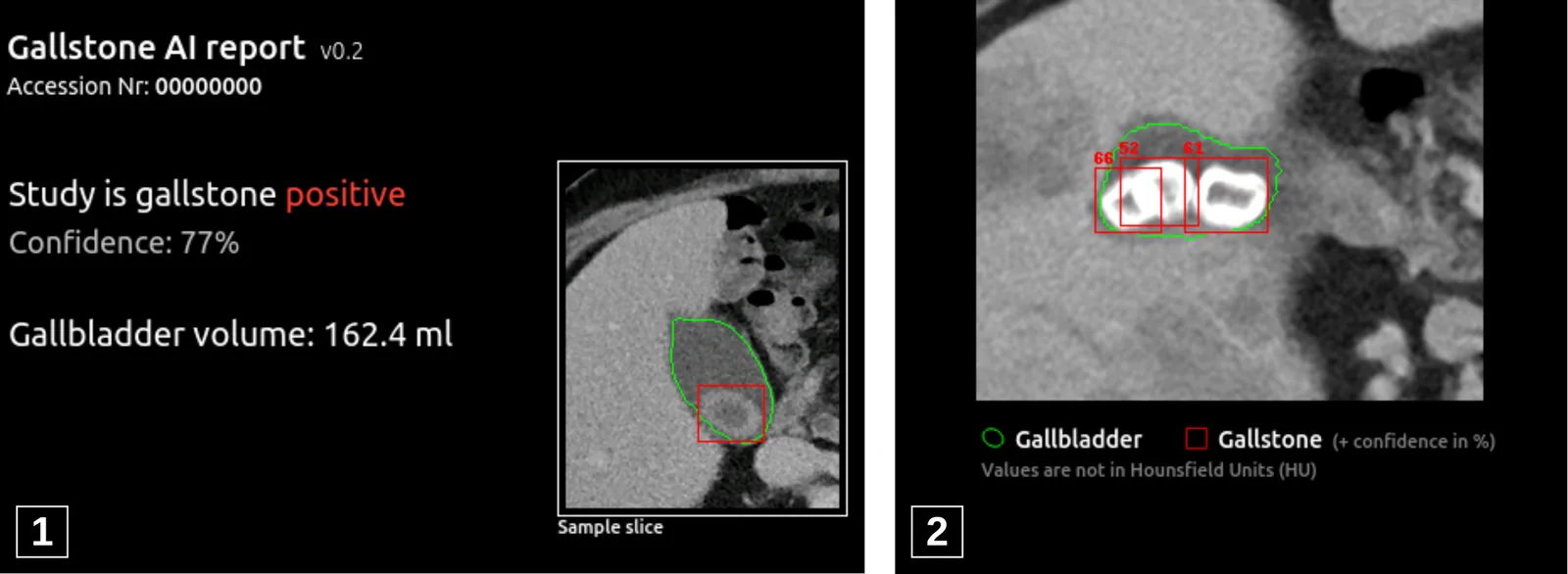
Examples of two different gallstone reports generated using results from the trained end-to-end algorithm. The report is converted to DICOM-format and can be sent to the hospital’s PACS. **a** Overview page with gallstone findings, gallbladder volume and a sample slice (gallstone with the highest detection confidence). **b** Scrolling further down shows the original CT data with bounding box overlays for gallstones (red) and gallbladder segmentation boundaries (green)

A few examples of the algorithm’s predictions are displayed in Fig. 4. The final gallstone report of a CT scan, which can be sent to the picture archiving and communication system (PACS) in the digital imaging and communications in medicine (DICOM) format, is illustrated in Fig. 5.

### Correlation with US and MRI findings

Of 963 included CT studies with a gallbladder, 178 (18.5%) had at least one ultrasound or MRI exam performed within 6 months of the index CT scan. Of these, one CT scan (0.6%) was false-positive and eight (4.5%) were false-negative for cholecystolithiasis compared to the findings on ultrasound or MRI reports. Additionally, 22 patients (15.9%) showed conflicting results across the three modalities.

## Discussion

The results of this study indicate that cholecystolithiasis can be automatically detected with high diagnostic accuracy on CT scans using standard deep learning techniques. The dataset comprised a representative population from a tertiary care hospital and included non-trivial cases. The algorithm excelled at detecting highly conspicuous gallstones, achieving a sensitivity > 97% for hyperdense and large stones, and maintained respectable performance for detecting less conspicuous gallstones, often challenging even for a human reader. As an ancillary discovery, we observed that patients with cholecystolithiasis had significantly larger gallbladder volumes than those without, suggesting a potential area for further investigation.

A common challenge in applying deep learning to medical imaging is data scarcity, particularly for rare cases. To increase the algorithm’s exposure to rarer gallstone types, such as hypodense and gaseous stones, we augmented the training data for some models by randomly pasting gallstone images from one patient into the gallbladder of others, thereby improving overall sensitivity. Training with three consecutive slices enhanced the specificity by providing spatial context. Finally, each slice is evaluated by multiple object detection algorithms, each specializing in different aspects. The final prediction is derived from a consensus measure (ensembling), which notably improved the algorithm’s performance.

To the best of our knowledge, this study is the first to assess the diagnostic accuracy of a deep learning algorithm for detecting cholecystolithiasis on CT scans. In related research, Song et al. used a different approach by training a deep learning model for gallstone segmentation (as opposed to gallstone object detection in our study) and reported a Dice score of 87% [32]. Similarly, Pang et al. used a YOLOv3-based object detection algorithm on a CT dataset of 1369 patients with cholecystitis and gallstones in a Chinese population, all of whom later underwent cholecystectomy [33]. They achieved a mAP of 86.5% for gallstone detection, compared to 81.7% in our study; however, the specifics of their mAP calculation were not detailed. Our study differs as it excludes patients with cholecystitis, complicating direct comparisons. Furthermore, since large gallstones (> 10 mm) are associated with a higher risk of acute cholecystitis [2], Pang et al.’s cohort likely had a higher proportion of large stones, for which our algorithm showed comparatively high sensitivity.

Detection of gallstones on CT scans is generally considered an easy task. However, a review of the literature on incidental findings on CT scans suggests that gallstones are often underreported [11–17], especially in trauma patients. In our experience, underreporting is typically due to time pressure, the incidental nature of the finding, and lower attention to non-primary regions. The output of our algorithm can be used to automatically add standardized text to the radiology report, indicating whether the gallbladder is present or absent (post-cholecystectomy) and whether gallstones are detected. This may support more consistent reporting of incidental cholecystolithiasis in a highly time-efficient and unobtrusive manner. We also developed an automatically generated summary of the algorithm’s results, which can be sent as a dedicated DICOM series to the PACS for direct visualization (Fig. 5). Additional potential applications include automated calculation of gallbladder volume, high-throughput screening of large imaging databases for research purposes, and use as an intuitive decision-support element for referring physicians.

Key limitations of this study include: First, the diagnostic gold standard for gallstone detection involves cholecystectomy and pathological examination, which suggests that the CT-based diagnostic performance reported here might be overestimated or biased due to the inherent limitations of CT imaging. To assess misclassification rates, we collected additional ultrasound and MRI reports of the same patients where available. Since only a few CT studies yielded false positives (0.6%) or false negatives (4.5%), we assume that the bias in our findings is low. Furthermore, the primary goal was to reduce missed detections caused by human oversight, rather than to improve overall detection rates on CT. Second, we were unable to validate our method externally due to the absence of a publicly available dataset with labelled gallstones on CT images. This monocentric study’s findings may not be generalizable to other populations with different gallstone compositions or using different CT protocols and scanners. Third, the dataset consisted exclusively of portal venous phase contrast-enhanced CT, reducing the model’s generalizability to other protocols. Non-contrast or non-portal-venous acquisitions at our institution are typically only performed for focused indications, and the gallbladder or liver are frequently only partly included. In addition, the standard reporting template for these studies does not routinely document the presence of gallstones. For these reasons, only portal venous scans were used for model development. Accordingly, diagnostic performance on non-contrast and non-portal-venous phase CT cannot be inferred from the present results. Fourth, while the algorithm is expected to perform well on familiar cases, it may misclassify unusual findings, such as post-surgery anatomy or malignancies. Fifth, the cholecystectomy detection subtask showed a sensitivity of only 76.7% and struggled with post-operative anatomy, particularly in the presence of fluid collections or surgical clips. Finally, the sensitivities for different gallstone types were assessed on validation splits, not the test set, due to the limited number of some stone types. This potentially biases results but should nonetheless provide an overview of the algorithm’s strengths and weaknesses.

## Data Availability

The data supporting the findings of this study are not publicly available due to data sensitivity and privacy restrictions imposed by the institutional ethics committee. Data may be made available from the corresponding author upon reasonable request. The data are stored in a controlled-access repository at our institution.

## Abbreviations

AUROC: Area under the receiver operating characteristic curve
CI: Confidence interval
DICOM: Digital imaging and communications in medicine
EMA: Exponential moving average
IoU: Intersection over union
IQR: Interquartile range
mAP: Mean average precision
PACS: Picture archiving and communication system
RIS: Radiology information system
ROC: Receiver operating characteristic
